# Proteome-Wide Identification of Lysine Succinylation in the Proteins of Tomato (*Solanum lycopersicum*)

**DOI:** 10.1371/journal.pone.0147586

**Published:** 2016-02-01

**Authors:** Weibo Jin, Fangli Wu

**Affiliations:** College of Life Sciences, Zhejiang Sci-Tech University, Hangzhou, 310018, China; National Taiwan University, TAIWAN

## Abstract

Post-translational modification of proteins through lysine succinylation plays important regulatory roles in living cells. Lysine succinylation was recently identified as a novel post-translational modification in *Escherichia coli*, yeast, *Toxoplasma gondii*, HeLa cells, and mouse liver. Interestingly, only a few sites of lysine succinylation have been detected in plants to date. In this study, we identified 347 sites of lysine succinylation in 202 proteins in tomato by using high-resolution mass spectrometry. Succinylated proteins are implicated in the regulation of diverse metabolic processes, including chloroplast and mitochondrial metabolism. Bioinformatic analysis showed that succinylated proteins are evolutionarily conserved and involved in various cellular functions such as metabolism and epigenetic regulation. Moreover, succinylated proteins exhibit diverse subcellular localizations. We also defined six types of definitively conserved succinylation motifs. These results provide the first in-depth analysis of the lysine succinylome and novel insights into the role of succinylation in tomato, thereby elucidating lysine succinylation in the context of cellular physiology and metabolite biosynthesis in plants.

## Introduction

Chromatin has a dynamic multi-level organization starting from the nucleosomal basic unit to the formation of a 30 nm fiber followed by high-order folding, which forms chromosomes [1]. Nucleosome remodeling, histone post-translational modifications (HPTMs), DNA methylation, and other factors define various chromatin states that drive transcription and other chromatin-based nuclear processes [2–4]. In particular, HPTMs largely regulate transcription and participate in DNA replication, histone deposition, and DNA repair and recombination. HPTMs occurring in core histone tails involve various covalent modifications, including acetylation, methylation, phosphorylation, ubiquitination [2], and succinylation [5, 6]. Lysine succinylation is a post-translational modification where a succinyl group is added to a lysine residue of a protein molecule.

Among amino acids, lysine is a frequent modification target because it defines the spatial structure of proteins, which in turn regulates protein functions. Mounting evidence indicates that lysine post-translational modification (PTM), which includes methylation [7], ubiquitination [8], acetylation [9–10], and succinylation [5, 6], is an efficient biological mechanism for both broadening and controlling protein function. In contrast with both lysine methylation and acetylation, lysine succinylation promotes more substantial transformation of the chemical properties of proteins owing to the transfer of a large structural moiety. Importantly, succinylation of a lysine residue induces transformation of charge status from +1 to −1 under certain physiological pH conditions [11], which in turn facilitates the structural adjustments and modifications in the functions of substrate proteins. Consequently, further conspicuous structural alteration owing to lysine succinylation can possibly promote more remarkable changes in protein structure and function.

Lysine succinylation (Ksucc) has been widely investigated and validated in various organisms, including bacteria (*Escherichia coli*), fungi (yeast), protozoan and parasite (*Toxoplasma gondii*), as well as mammalian cells (human and mouse) [7, 11–13]; however, little is known regarding Ksucc in plants. In this study, systematic identification of the lysine succinylome of tomato was done using an integrated proteome-wide method. Overall, we identified 347 unique lysine succinylation sites common in 202 succinylated proteins with diverse cellular localizations and biological functions. Six unique motifs were also found through bioinformatic analysis of the sequences flanking the succinylation sites. To our knowledge, these results provide the first comprehensive analysis of the tomato succinylome.

## Materials and Methods

### Reagents

The reagents, namely trichloroacetic acid (TCA), dithiothreitol (DTT), iodoacetamide (IAA), trifluoroacetic acid (TFA), ammonium bicarbonate (NH_4_HCO_3_) and ammonium formate, were all purchased from Sigma-Aldrich (St. Louis, MO, USA). Acetonitrile (ACN) and pure water were obtained from Thermofisher (Waltham, MA, USA), while 2-D Quant kit was obtained from GE Healthcare (Buckinghamshire, United Kingdom). Trypsin was purchased from Promega (Fitchburg, WI, USA) and the protease inhibitor cocktail set IV was obtained from Millipore (Billerica, MA, USA). The anti-succinyllysine antibody agarose conjugated beads were obtained from PTM Biolabs (Hangzhou, China) and Sep-Pak C18 SPE columns were purchased from Waters (Framingham, USA).

### Protein Extraction, Trypsin Digestion and HPLC Fractionation

Tomatoes (*S*. *lycopersicum*) cv. micro-Tom were grown in a greenhouse at a 16-h day/8-h night cycle, at 22–28°C. At the age of 2 months, similar quantities of the roots, stems, and leaves were obtained and mixed at 11:00 am. The mixture was ground after immersing in liquid nitrogen, and the resulting powder was lysed three times in a cold solution containing 8 M urea, 1% Triton-100, 65 mM DTT, and 1% protease inhibitor cocktail set IV using a high-intensity ultrasonic processor (Scientz, Ningbo, China). Unbroken debris were removed by centrifugation at 20,000x *g* for 10 min at 4°C. Proteins in lysis buffer were then precipitated with cold 15% TCA at −20°C for 2 h. After centrifugation at 5,000x *g* for 10 min at 4°C, the precipitate was washed three times with cold acetone and redissolved in buffer containing 8 M urea and 100 mM NH_4_HCO_3_ at pH 8.0. Finally, the protein concentration in the supernatant was determined using 2-D Quant kit according to the manufacturer’s protocol.

For digestion, the protein solution was reduced using 10 mM DTT for 1 h at 56°C and then alkylated in darkness with 20 mM IAA for 45 min at room temperature. After that, the solution was diluted with ammonium bicarbonate to urea concentration less than 2 M. Finally, trypsin was added at 1:50 trypsin-to-protein mass ratio overnight for the first digestion step and at 1:100 for 4 h for the second digestion step. The peptide solution thus obtained was desalted with Sep-Pak SPE column and lyophilized to dryness.

To enhance the accuracy and throughput of protein identification, proteins were fractionated by high pH reverse-phase HPLC using an Agilent 300Extend C18 column (5 μm particles, 4.6 mm ID, 250 mm length) along with solvent A (98% H_2_O and 2% acetonitrile containing 10 mM ammonium formate, pH 10) and solvent B (2% H_2_O and 98% acetonitrile containing 10 mM ammonium formate). The LC gradient was run with 2% to 60% solvent B for 80 min to generate 80 fractions at 1 min per fraction, after which all fractions were combined into 8 fractions. The fractionated sample was dried by vacuum centrifugation and stored at −20°C.

### Affinity Enrichment of Lysine Succinylated Peptides

To enrich succinylated peptides, the fractionated tryptic peptides were re-dissolved in NETN buffer (100 mM NaCl, 1 mM EDTA, 50 mM Tris-HCl, 0.5% NP-40, pH 8.0) and incubated with anti-succinyllysine antibody agarose conjugated beads (PTM Biolabs, Hangzhou, China) at a ratio of 15 μL beads/mg protein at 4°C overnight with gentle shaking. After incubation, the beads were washed four times with NETN buffer and twice with pure water. The bound peptides were eluted with 1% TFA and dried under vacuum.

### LC-MS/MS Analysis

Peptides were dissolved in solvent A (0.1% FA in 2% ACN, 98% H_2_O), directly loaded onto a reversed-phase pre-column (Acclaim PepMap100 C18 column, 3 μm, 75 μm × 2 mm, 100 Å, Thermo Scientific). Peptide separation was performed using a reversed-phase analytical column (Acclaim PepMap RSLC C18 column, 50 μm × 15 mm, 2 μm, 100 Å, Thermo Scientific). The gradient was comprised of an increase from 6% to 22% solvent B (0.1% FA in 98% ACN) for 24 min, 22% to 36% for 10 min and climbing to 80% in 3 min then holding at 80% for the last 3 min, at a constant flow rate of 300 nl/min on an EASY-nLC 1000 UPLC system. The resulting peptides were analyzed using a Q Exactive^™^ Plus hybrid quadrupole-Orbitrap mass spectrometer (Thermo Scientific).

The peptides were subjected to NSI source followed by tandem mass spectrometry (MS/MS) in Q Exactive^™^ Plus coupled online to the UPLC. Intact peptides were detected in the Orbitrap at a resolution of 70,000. Peptides were selected for MS/MS using NCE setting of 28; ion fragments were detected in the Orbitrap at a resolution of 17,500. A data-dependent procedure that alternated between one MS scan followed by 20 MS/MS scans was applied for the top 20 precursor ions above a threshold ion count of 1.5E4 in the MS survey scan with 15.0 s dynamic exclusion. The electrospray voltage applied was 2.0 kV. Automatic gain control (AGC) was used to prevent overfilling of the ion trap; 5E4 ions were accumulated for generation of MS/MS spectra. For MS scans, the m/z scan range was 350 to 1800.

### Database searching

Protein and succinylation sites were identified using MaxQuant along with the integrated Andromeda search engine (v. 1.4.1.2). The MS/MS data was searched against the Lycopersicon esculentum protein subset in the Uniprot database (34,824 sequences, http://www.ebi.ac.uk/uniprot/) and concatenated with a reverse decoy database. During the MaxQuant database searches, trypsin/P was specified as the enzyme, with up to four missed cleavages allowed. Additional parameters included a maximum of five modifications per peptide and charge states of up to 5. The mass error was set to 4.5 ppm for the precursor ions and 0.02 Da for the fragment ions. Moreover, carbamidomethylation (+57.0215 Da) on cysteine was specified as a fixed modification, whereas oxidation (+15.9949 Da) on methionine, succinylation (+100.0160 Da) on lysine and acetylation (+42.0106 Da) on protein N-terminus were specified as variable modifications. Furthermore, false discovery rate thresholds were specified at 0.01 for modification sites, peptides, and proteins. The minimum length of peptide was set at 7 amino acids. Succinylation site identifications with localization probability less than 0.75 or from reverse sequences were removed.

### Bioinformatic analysis

#### Protein Annotation

Gene Ontology (GO) annotation of the proteome was done using the UniProt-GOA database (http://www.ebi.ac.uk/GOA/). When a single peptide was found to match two or more different proteins, manual inspection of the data was performed to determine the protein from which the peptide was likely derived.

#### KEGG Pathway Annotation

Kyoto Encyclopedia of Genes and Genomes (KEGG) database was used to annotate the protein pathway. First, a KEGG online service tool, KAAS, was used to annotate the protein’s KEGG database description. Subsequently, the annotation result was mapped on the KEGG pathway database by using another KEGG online service tool, the KEGG mapper. Wolf PSORT software was employed to predict subcellular localization.

#### GO/KEGG Pathway Functional Enrichment Analysis

For enrichment or depletion (right-tailed test) of specific annotation terms among the members of resulting protein clusters, Fisher’s exact test was used to obtain the p values. In all of the clusters, any terms with p values below 0.05 were treated as significant.

#### Analysis of Sequence Model around Succinylation Sites

The sequence models consisted of amino acids at specific positions of the succinyl-21-mers (10 amino acids upstream and downstream of the succinylation sites) in all protein sequences surveyed using Motif-x. Additionally, the entire tomato database of protein sequences was used as background database parameter, and other parameters were set as default.

#### Motif-Based Clustering Analysis

All lysine succinylation substrate categories obtained after enrichment were collated along with their p values and then filtered for categories that were at least enriched in one of the clusters with p value < 0.05. This filtered p-value matrix was transformed using the function x = −log 10 (p value). Finally, these *x* values were *z* transformed for each category. Subsequently, these *z* scores were clustered by one-way hierarchical clustering (Euclidean distance, average linkage clustering) in Genesis. Cluster membership was visualized by plotting a heat map using the “heatmap.2” function in the “ggplots” R-package.

## Results and Discussion

### LC–MS/MS Analysis of Succinylated Lysine Peptides in Tomato

**Fig 1:**
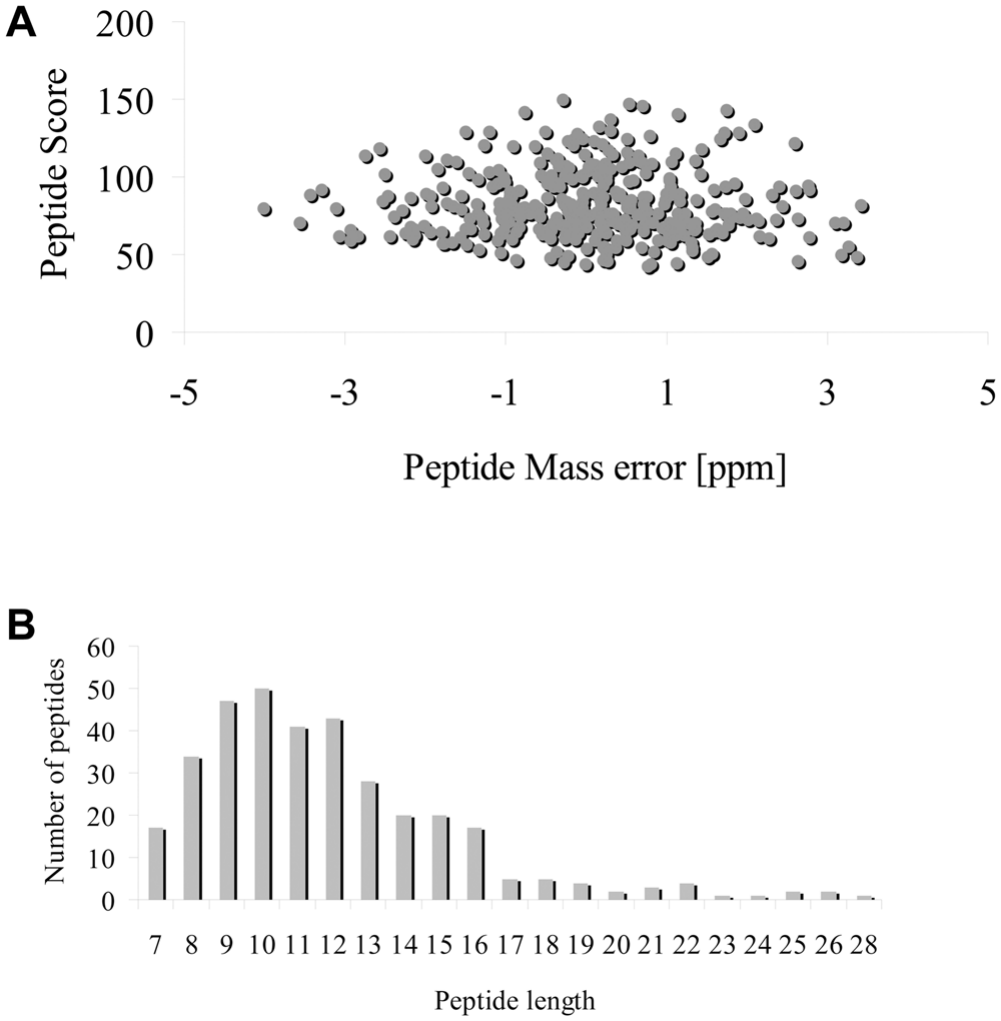
Evaluation of the quality of MS and MS/MS data. (A) Peptide score of all succinylated peptides is plotted as a function of calibrated peptide mass errors measured from all identified peptides in parts-per-million (ppm). (B) Length distribution of the identified peptides.

Lysine succinylation is a new acylation type of PTM that can regulate protein function in both prokaryotic and eukaryotic cells in diverse ways [14]. However, the succinylome of plants has not been reported to date. Thus, in order to obtain a detailed view of lysine succinylation sites in plants, proteins were isolated from a mixed tomato sample with equal roots, leaves, and stems. After digestion with trypsin, succinylated peptides were enriched by affinity purification with succinyl-lysine antibodies. Subsequently, the enriched succinylated peptides were surveyed by LC–MS/MS. Fig 1 shows the data obtained from high-quality LC–MS/MS. We found that the mass error of most of the identified peptides was nearly zero, mostly <0.02 Da, indicating accuracy of the MS data (Fig 1A). In addition, the length of most identified peptides ranged from 7 to 20, which is consistent with the known property of tryptic peptides (Fig 1B). Thus, the sample preparation method and LC–MS/MS data met approval standards. All MS data have been deposited in Proteome Xchange (Project accession: PXD002380).

### Proteome-Wide Mapping of Lysine Succinylation Sites

The obtained LC–MS/MS data were matched with the *L*. *esculentum* database (with a total of 34,824 sequences) concatenated with reverse decoy database. A total of 347 lysine succinylation peptides with peptide score >40 (S1 Table) were identified in tomato. These peptides with varying abundance depending on their length occurred on 202 succinylated proteins with different numbers of succinylated sites ranging from 1 to 9. Out of the 202 identified succinylated proteins, 60.4% (122/202) had a single succinylated site, 24.6% (49/202) had two, and 8.4% (17/202) contained three; the average degree of succinylation was 1.7 (347/202). Notably, most of the proteins with multiple succinylations were chloroplast and mitochondrial proteins involved in diverse metabolic pathways (S1 Table). Moreover, the most extensively succinylated protein with up to nine independent lysine residues was dihydrolipoyl dehydrogenase, which is a mitochondrial enzyme and plays a vital role in energy metabolism (S1 Table).

### Characterization of Lysine Succinylome in Tomato

GO functional classification was performed on succinylated proteins in tomato to reveal the biological processes they were involved in. The results revealed that succinylation occurred in proteins associated with a diverse range of biological processes, cellular components, and molecular functions. These findings indicated that succinylation is an important PTM in tomato (Table 1). On the basis of biological processes, the largest class of succinylated proteins (27%) participated in metabolic processes. The second largest class accounted for 23% of total proteins and consisted of proteins involved in cellular processes. On the basis of molecular function, the largest class of succinylated proteins, accounting for 44% of the total, consisted of binding proteins. The second largest class, accounting for 43%, was comprised of proteins involved in catalytic activities (Table 1). These findings are consistent with those of previous studies on bacteria and eukaryotic cells [14], suggesting the essential regulatory roles of succinylated proteins in cells.

**Table 1:** GO classification for lysine succinylated protein.

	Class name	Number of members
Biological Process	metabolic process	158
	cellular process	132
	single-organism process	116
	response to stimulus	69
	localization	20
	cellular component organization or biogenesis	16
	multi-organism process	14
	developmental process	13
	multicellular organismal process	12
	biological regulation	12
	other	11
Molecular Function	binding	125
	catalytic activity	122
	transporter activity	16
	electron carrier activity	9
	molecular function regulator	6
	other	6

**Fig 2:**
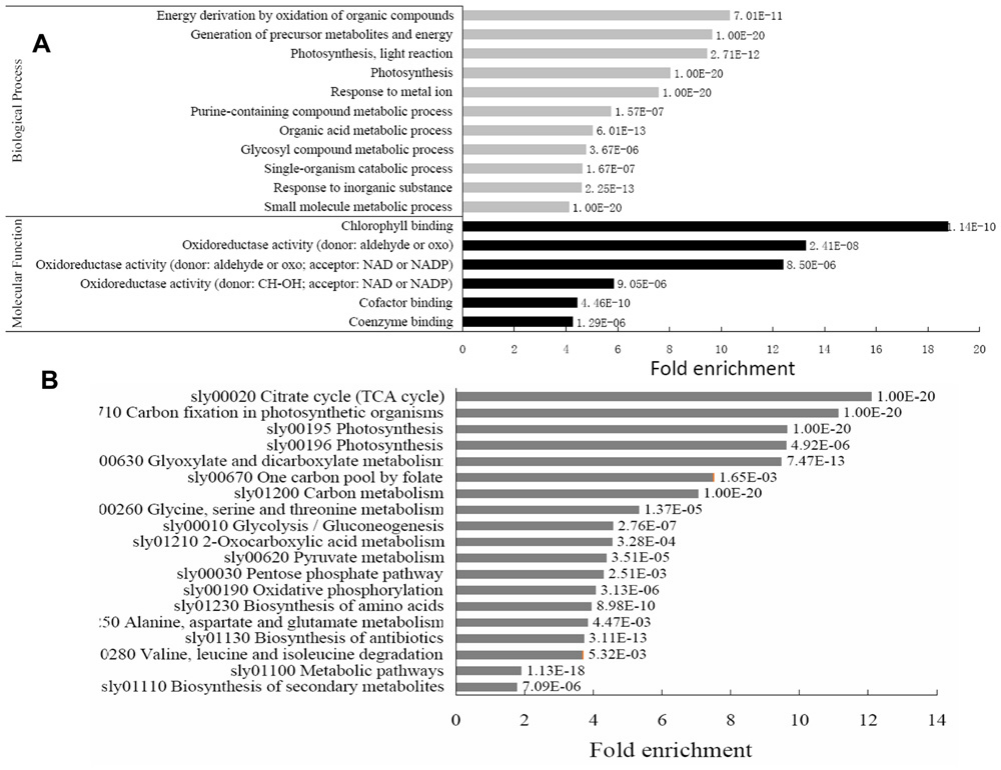
Characterization of lysine succinylome in tomato. Enrichment analysis of succinylated proteins according to the classification of GO annotation, which is based on biological processes (gray bars) (p < 0.05) and molecular function (black bars) (A) and KEGG pathway enrichment analysis (p < 0.05) (B).

We also evaluated the enrichment of succinylation sites to determine which functional categories are the preferred targets of lysine succinylation. A wide range of metabolic processes such as photosynthesis, energy derivation, single-organism carbohydrate metabolism were significantly enriched (Fig 2A, S2 Table). Moreover, based on GO molecular functions, the succinylated proteins involved in chlorophyll binding and oxidoreductase activity were the most enriched (Fig 2A, S2 Table). These results suggested that protein succinylation is an essential regulatory mechanism in photosynthesis and oxidation–reduction reactions in tomato.

We also performed KEGG enrichment analysis for a more comprehensive understanding of the metabolic processes in tomato (Fig 2B, S3 Table). Results showed that multiple metabolic pathways, such as photosynthesis, citrate cycle, and carbon metabolism, were highly represented in the tomato succinylome, indicating a vital role of lysine succinylation in most of the fundamental cellular processes in tomato. Both carbon fixation and citrate cycle were also enriched by more than 10-fold, validating that lysine succinylation regulates photosynthesis and respiratory metabolism.

### Subcellular Localization and Pathway Analysis of Succinylated Proteins in Tomato

**Fig 3:**
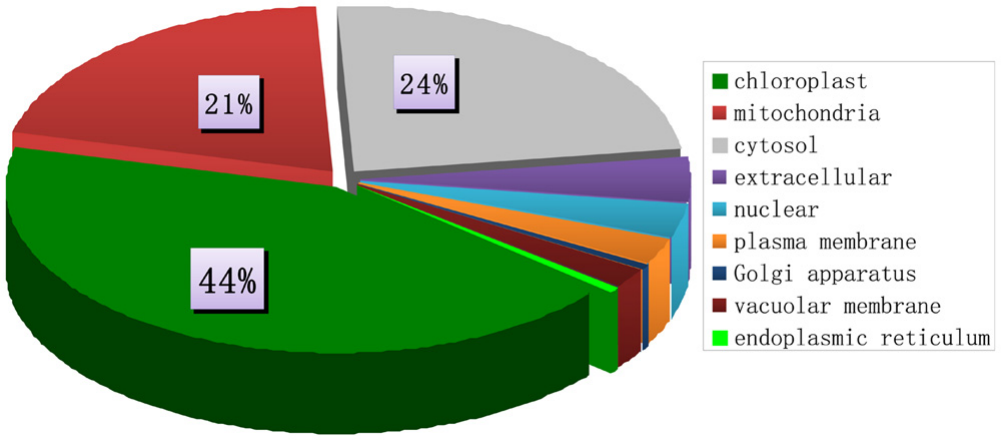
Subcellular location of lysine succinylome of tomato.

Identifying the localization of proteins is important to elucidate their interactions with other molecules, as well as their biological functions. Therefore, we predicted the subcellular locations of succinylated proteins by using Wolf PSORT software. Our results showed for the first time that the largest class of succinylated proteins was located in the chloroplast, accounting for ~44% of the 202 succinylated proteins in tomato (Fig 3). Pathway enrichment analysis through the KEGG database also showed that lysine succinylation occurred on several subunits of nearly every protein complex involved in photosynthesis, such as PsbD/C/B and PsbO/P/Q/R/S in photosystem II, PsaA/C/D/E/H in photosystem I, PetD/H on phytochrome b6/f complex, PetH in photosynthethic electron transport, and β/α/γ/c/b on ATP synthase, Lhca1/3, and Lhcb1/2/4/5/6 in light-harvesting chlorophyll protein complex (Table 2, S1 and S2 Figs). These results suggested that succinylation in chloroplast proteins regulates photosynthesis.

**Table 2:** Succinylated proteins involved in photosynthesis pathway.

Protein Acc.	KEGG annotation
**sly00195 Photosynthesis**	
K4C7M7; Q2MIB4	ATPF0B: F-type H+-transporting ATPase subunit b
Q2MIB3	ATPF0C: F-type H+-transporting ATPase subunit c
Q2MIB5	ATPF1A: F-type H+-transporting ATPase subunit alpha [EC:3.6.3.14]
Q2MI93	ATPF1B: F-type H+-transporting ATPase subunit beta [EC:3.6.3.14]
K4B9S5	ATPF1G: F-type H+-transporting ATPase subunit gamma
Q2MI87	petA: apocytochrome f
Q2MI70	petD: cytochrome b6-f complex subunit 4
K4B6A3; K4BAP9	petH: ferredoxin—NADP+ reductase [EC:1.18.1.2]
Q2MIA0	psaA: photosystem I P700 chlorophyll a apoprotein A1
Q2MI49	psaC: photosystem I subunit VII
P12372	psaD: photosystem I subunit II
K4CU43	psaE: photosystem I subunit IV
K4DFB6	psaH: photosystem I subunit VI
Q2MI75	psbB: photosystem II CP47 chlorophyll apoprotein
Q2MIA4	psbC: photosystem II CP43 chlorophyll apoprotein
Q2MIA5	psbD: photosystem II P680 reaction center D2 protein
K4BCF4	psbO: photosystem II oxygen-evolving enhancer protein 1
P29795	psbP: photosystem II oxygen-evolving enhancer protein 2
Q40163	psbR: photosystem II 10kDa protein
P54773	psbS: photosystem II 22kDa protein
Q672Q6	psbQ: photosystem II oxygen-evolving enhancer protein 3
**sly00196 Photosynthesis—antenna proteins**	
P12360	LHCA1: light harvesting complex I chlorophyll a/b binding protein 1
P27522	LHCA3: light harvesting complex I chlorophyll a/b binding protein 3
P07369	LHCB1: light harvesting complex II chlorophyll a/b binding protein 1
P14278	LHCB2: light harvesting complex II chlorophyll a/b binding protein 2
K4CRS9	LHCB4: light harvesting complex II chlorophyll a/b binding protein 4
K4C768	LHCB5: light harvesting complex II chlorophyll a/b binding protein 5
P27525	LHCB6: light harvesting complex II chlorophyll a/b binding protein 6

Succinyl-CoA and succinate are mainly derived from the mitochondria during tricarboxylic acid cycle or odd-numbered fatty acid oxidation. Succinylation of proteins thus occurs on more mitochondrial proteins of some eukaryotic organisms. Succinylation of mitochondrial proteins was recently reported to occur in yeast, *Toxoplasma*, HeLa cells, and mouse liver in approximately 8%, 26%, 45%, and 70% of proteins, respectively [13, 14]. In the present study, we found that succinylation occurred in approximately 21% (41/202) of mitochondrial proteins of tomato, which is a lower proportion compared to other eukaryotic organisms. Moreover, KEGG pathway enrichment analysis revealed that nearly every enzyme involved in the tricarboxylic acid cycle was succinylated, suggesting an important role of succinylation in the TCA cycle (Table 3, S3 Fig). These findings are consistent with those of Weinert et al. and Li et al. [13, 14].

**Table 3:** Succinylated proteins involved in tricarboxylic acid cycle.

Protein Acc.	KEGG annotation
K4CW40	MDH1: malate dehydrogenase [EC:1.1.1.37]
K4CGU8	MDH2: malate dehydrogenase [EC:1.1.1.37]
K4D1P2; K4CNF2	IDH3: isocitrate dehydrogenase (NAD+) [EC:1.1.1.41]
K4BBG9	IDH1: isocitrate dehydrogenase [EC:1.1.1.42]
K4BWH8	PDHA: pyruvate dehydrogenase E1 component alpha subunit [EC:1.2.4.1]
K4C8X8	PDHB: pyruvate dehydrogenase E1 component beta subunit [EC:1.2.4.1]
K4BPJ0	OGDH: 2-oxoglutarate dehydrogenase E1 component [EC:1.2.4.2]
D2KQI9	SDHB: succinate dehydrogenase (ubiquinone) iron-sulfur subunit [EC:1.3.5.1]
Q8GT30	DLD: dihydrolipoamide dehydrogenase [EC:1.8.1.4]
K4D533; K4CBF0	DLAT: pyruvate dehydrogenase E2 component (dihydrolipoamide acetyltransferase) [EC:2.3.1.12]
K4DB46; K4CHF9	DLST: 2-oxoglutarate dehydrogenase E2 component (dihydrolipoamide succinyltransferase) [EC:2.3.1.61]
K4DCI6; K4AXC0	CS: citrate synthase [EC:2.3.3.1]
K4CFD4	ACO: aconitate hydratase [EC:4.2.1.3]
Q8GTQ9	LSC1: succinyl-CoA synthetase alpha subunit [EC:6.2.1.4 6.2.1.5]
Q84LB6	LSC2: succinyl-CoA synthetase beta subunit [EC:6.2.1.4 6.2.1.5]

### Lysine Succinylation of Histone Proteins in Tomato

**Fig 4:**
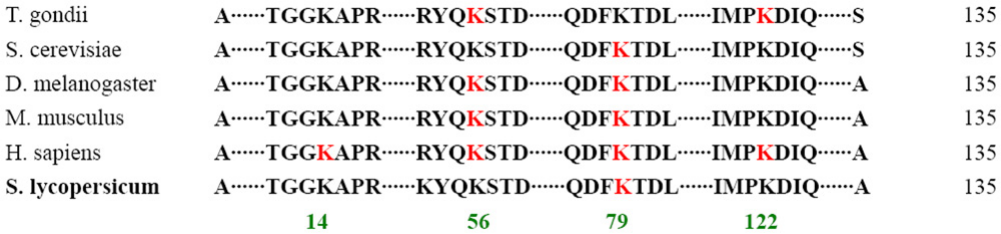
Comparison of the succinylated residues in histone H3 of *S*. *lycopersicum* with that of five other species. Red sequences indicate succinylation sites. Numbers below the sequences represent the amino acid position.

Previous studies demonstrated that histone modifications are involved in regulating gene transcription during plant growth and development, as well as in plant’s response to various endogenous and exogenous stimuli [15, 16]. To date, histone succinylation has been reported in several organisms, including bacteria, yeast, and animals, but not in plants. In the current study, we found that succinyl modification occurred only at histone H3 K79. H3K79succ was also found in human, mouse liver, *Saccharomyces cerevisiae*, and *Drosophila melanogaster* (Fig 4), indicating that this succinylation site is conserved between animal and tomato. Additionally, lysine succinylation of histone H3 in tomato was not observed at other sites, namely, H3K14, H3K56, and H3K122, which occur in human, mouse liver, *S*. *cerevisiae*, *D*. *melanogaster*, and *T*. *gondii* (Fig 4).

Moreover, only one lysine site was succinylated in tomato histone H2B, and the site is different from that in human, mouse liver, and *T*. *gondii*, indicating that it is a tomato-specific H2B succinylation site (data not shown). Finally, no succinylation sites were detected in histone H4 and H2A. Possible explanations for these negative results are: (i) peptides corresponding to H4 and H2A were less abundant; (ii) these proteins were inadvertently destroyed during sample preparation; and (iii) acid extraction altered the accessibility of proteins.

### Motif Analysis of Identified Lysine Succinylated Peptides

**Fig 5:**
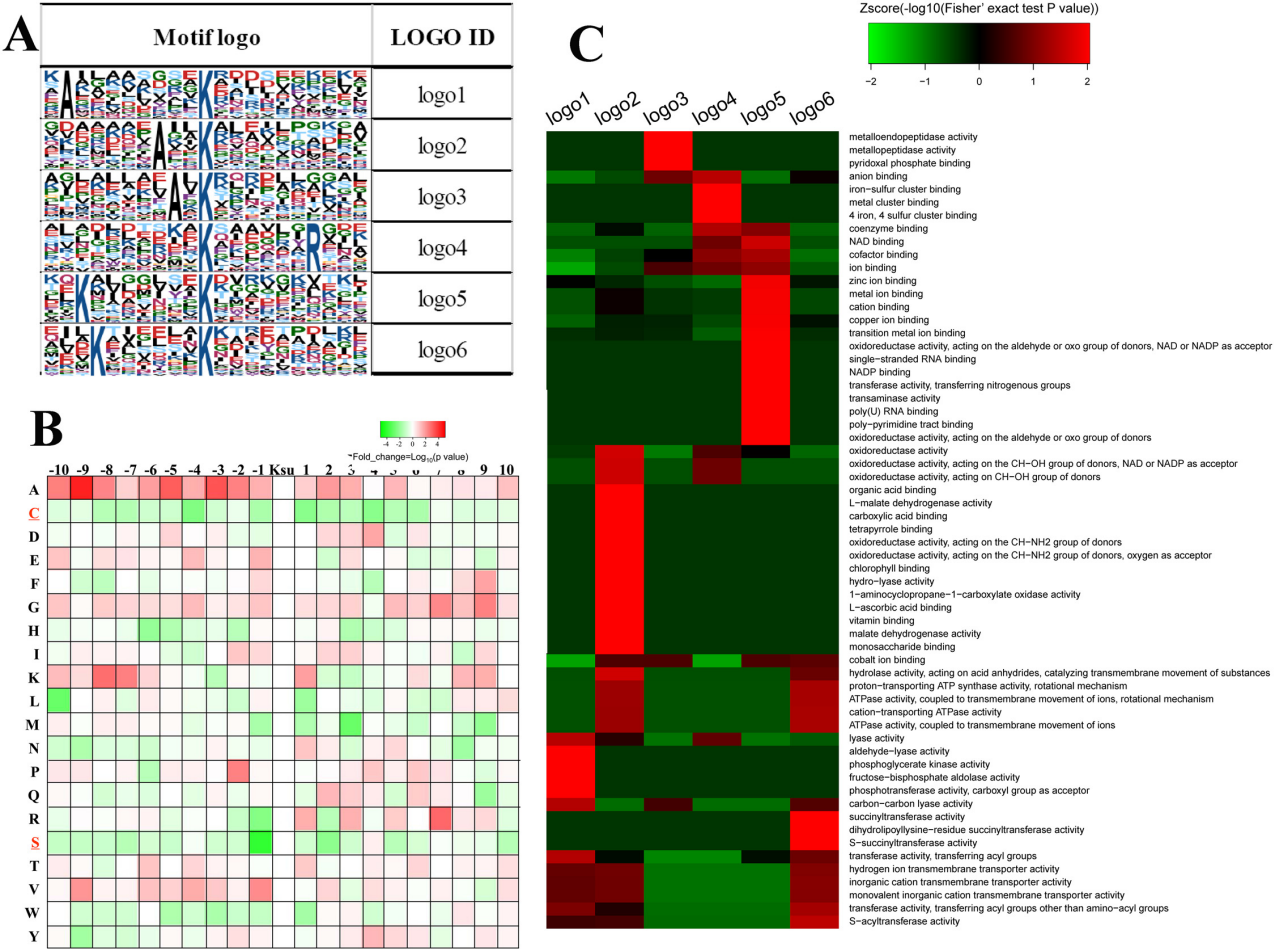
Characterization of succinylated peptides. (A) Motif-X revealed the probability sequence motifs of tomato succinylation sites consisting of 20 residues surrounding the targeted lysine residue. Six significantly enriched succinylation site motifs were identified. (B) Heat map showing enrichment (red) or depletion (green) of amino acids in specific positions flanking the succinylated lysine in Tomato. (C) Heat map showing enrichment (red) or depletion (green) of the six motifs in various proteins classified in terms of GO molecular function.

To evaluate the nature of succinylated lysine in tomato, we used Motif-X, which was developed to extract overrepresented patterns from any sequence, to identify the sequence motifs in all the identified succinylated lysines. Six definitively conserved succinylation site motifs were defined on the basis of 188 unique sites, accounting for 54.3% (188/347) of sites identified in terms of specific amino acid sequence located 10 amino acids upstream and downstream from the succinylated lysine (Fig 5A and Table 4). Furthermore, alanine residues were overrepresented in the -9, -3, and -2 positions of the succinylated sites, which were named logo1, logo2 and logo3, respectively. Arginine residues were overrepresented in the +7 position of the succinylated site, which was named logo4, while lysine residues were overrepresented in the -8 and -7 positions of the succinylated sites, which were named logo5 and logo6, respectively (Fig 5A and 5B; Table 4). In addition, cysteine (C) and serine (S) residues were underrepresented in the succinylated sites (Fig 5B).

**Table 4:** Motif Analysis for Identified the Succinylated Lysine Peptides in tomato.

Motif	Motif score	Foreground		background		Fold increase	Logo ID
		Matches	Size	Matches	Size		
.A........K..........	4.65	42	334	43530	684457	1.98	logo1
.....A..K..........	3.7	33	292	36992	640927	1.96	logo2
........A.K......	3.28	30	259	36482	603935	1.92	logo3
........K......R...	3.85	26	229	29126	567453	2.21	logo4
..K.....K.......	3.55	31	203	42192	538327	1.95	logo5
...K......K.......	3.27	26	172	37388	496135	2.01	logo6

A motif-based clustering analysis of succinylated proteins was also performed to visualize the function-specific sequence motifs. The results showed that most of logo1 motif contained proteins clustered into four enzyme activities, namely, aldehyde-lyase activity, phosphoglycerate kinase activity, fructose-bisphosphate aldolase activity, and phosphotransferase activity (Fig 5C). Most of the logo2 motifs were found in proteins clustered into oxidoreductase activity, whereas most of logo3-containing proteins clustered into metalloendopeptidase and metallopeptidase activities. Moreover, most proteins containing logo4 and logo5 clustered into binding activities, including ion binding, coenzyme binding, and RNA binding. Most of the proteins containing logo6 were enriched for succinyltransferase activity.

## Conclusions

By combining high-affinity enrichment of lysine-succinylated peptides with high-sensitivity mass spectrometry and bioinformatics tools, we conducted the first in-depth analysis of the lysine succinylome in plants, specifically tomato. We identified 347 lysine succinylation sites in 202 succinylated tomato proteins. Moreover, extensive characterization of the succinylome in tomato revealed that succinylation largely occurred in proteins involved in a broad range of functions, ranging from control of metabolic processes to biological regulation. Succinylated proteins were distributed across different cellular compartments, suggesting that protein succinylation is vital in regulating physiological processes in tomato. In particular, lysine succinylation of proteins related to photosynthesis was revealed for the first time, indicating other possible important roles in regulating physiological processes. Indeed, our data provide novel insights into the role of succinylation and the frequency of succinylation in tomato, thereby elucidating lysine succinylation in the context of cellular physiology and metabolite biosynthesis in plants.

## Supporting Information

S1 Fig Succinylated proteins involved in photosynthesis. Proteins in red are the succinylated proteins identified in this study.
(TIF)

S2 Fig Succinylated proteins involved in antenna proteins in photosynthesis. Proteins in red are the succinylated proteins identified in this study.
(TIF)

S3 Fig Succinylated proteins involved in the tricarboxylic acid cycle. Proteins in red are the succinylated proteins identified in this study.
(TIF)

S1 Table GO/KEGG annotation of succinylated protein in tomato. (XLS)

S2 Table GO enrichment analysis of the identified succinylated proteins in tomato. (XLS)

S3 Table KEGG pathway enrichment analysis of the identified succinylated proteins in tomato. (XLS)
