# Supplementary figures and images for: Proteome-Wide Identification of Lysine Succinylation in the Proteins of Tomato (Solanum lycopersicum)
Source: PLoS One. 2016 Feb 1;11(2):e0147586. doi: 10.1371/journal.pone.0147586 (PMC4734689; doi:10.1371/journal.pone.0147586)

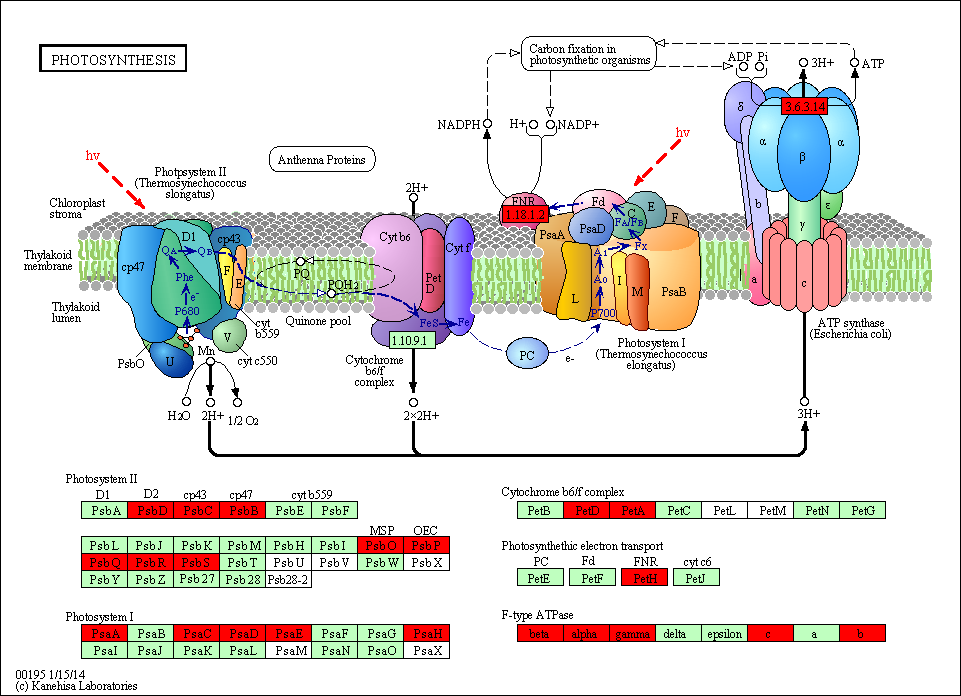

Supplement: S1 Fig — Proteins in red are the succinylated proteins identified in this study. (TIF) [file pone.0147586.s001.tif]

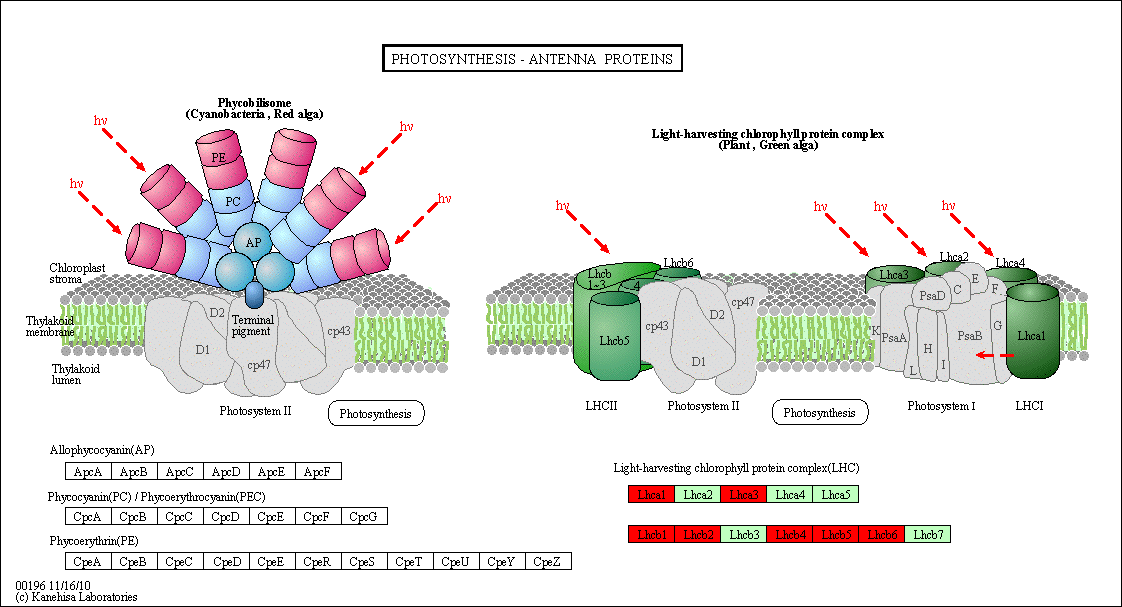

Supplement: S2 Fig — Proteins in red are the succinylated proteins identified in this study. (TIF) [file pone.0147586.s002.tif]

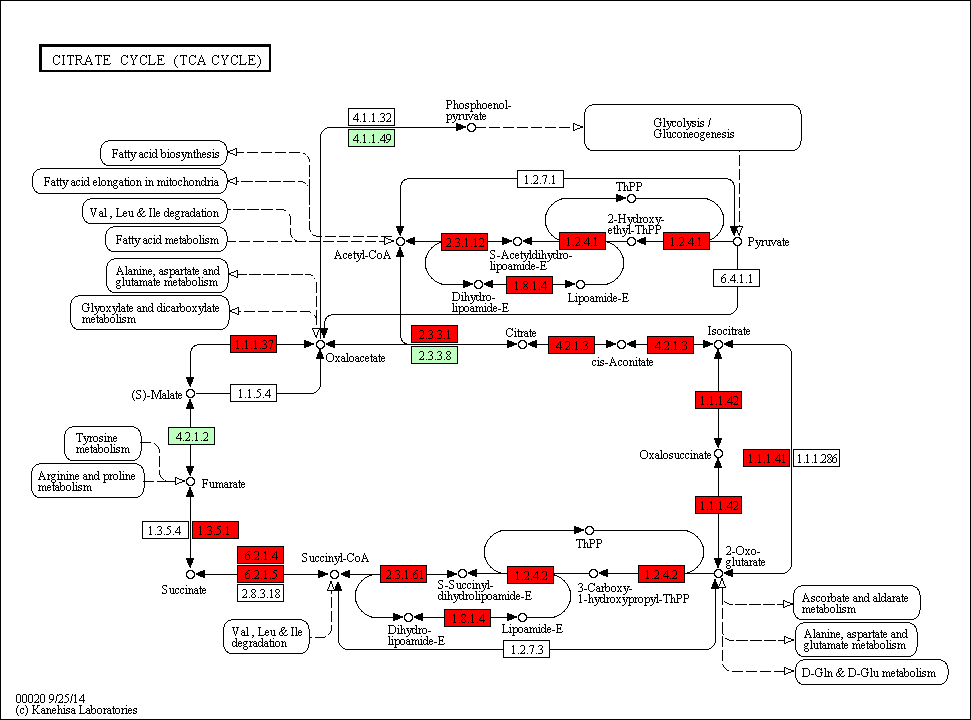

Supplement: S3 Fig — Proteins in red are the succinylated proteins identified in this study. (TIF) [file pone.0147586.s003.tif]
